# Metabolic Profile Changes of CCl_4_-Liver Fibrosis and Inhibitory Effects of Jiaqi Ganxian Granule

**DOI:** 10.3390/molecules21060698

**Published:** 2016-05-30

**Authors:** Ge Wang, Zehao Li, Hao Li, Lidan Li, Jian Li, Changyuan Yu

**Affiliations:** 1 College of Life Science and Technology, Beijing University of Chemical Technology, Beijing 100029, China; wanggebuct@163.com (G.W.); lizehao1990@126.com (Z.L.); lihao@buct.edu.cn (H.L.); cd_lilidan@163.com (L.L.); 2 School of Basic Medical Science, Beijing University of Chinese Medicine, Beijing 100029, China

**Keywords:** Jiaqi Ganxian granule, serum, liver fibrosis, metabolomics pathway, ultra-performance liquid chromatography-time-of-flight mass spectrometry

## Abstract

Jiaqi Ganxian Granule (*JGG*) is a famous traditional Chinese medicine, which has been long used in clinical practice for treating liver fibrosis. However, the mechanism underlying its anti-hepatic fibrosis is still not clear. In this study, an Ultra-Performance Liquid Chromatography-Time-Of-Flight Mass Spectrometry (UPLC-TOF-MS)-based metabolomics strategy was used to profile the metabolic characteristic of serum obtained from a carbon tetrachloride (CCl_4_)-induced hepatic fibrosis model in Sprague-Dawley (SD) rats with *JGG* treatment. Through Principal Component Analysis (PCA) and Partial Least Square Discriminant Analysis (PLS-DA), it was shown that metabolic perturbations induced by CCl_4_ were inhibited after treatment of *JGG*, for 17 different metabolites related to CCl_4_. Among these compounds, the change tendency of eight potential drug targets was restored after the intervention with *JGG*. The current study indicates that *JGG* has a significant anti-fibrosis effect on CCl_4_-induced liver fibrosis in rats, which might be by regulating the dysfunction of sphingolipid metabolism, glycerophospholipid metabolism, *N*-acylethanolamine biosynthesis, fat digestion and absorption, while glycerophospholipid metabolism played vital roles in the inhibitory effects of *JGG* on hepatic fibrosis according to Metabolic Pathway Analysis (MetPA). Our findings indicated that the metabolomics approach may provide a useful tool for exploring potential biomarkers involved in hepatic fibrosis and elucidate the mechanisms underlying the action of therapies used in traditional Chinese medicine.

## 1. Introduction

Hepatic fibrosis, caused by the imbalance of synthesis and degradation of Extracellular Matrix (ECM) [1], represents the wound healing response of the liver to repeated injury and involves a series of cell types and mediators [2]. It is a critical state in chronic liver disease, which remains a major medical problem with significant morbidity and mortality and can eventually develop into irreversible cirrhosis or liver cancer [3]. Therefore, the interruption and reversion of the hepatic fibrosis would be a potential therapeutic choice to prevent its progression [4]. Some synthetic drugs, such as *N*-acetylcysteine and 5-nitroso-*N*-acetylcysteine [5], can promote the degradation of fibrillar ECM and have been used to effectively treat hepatic fibrosis. However, these synthetic drugs are not natural products and may have some side effects. Antimalarials and related drugs (quinidine), sulfonamides (co-trimoxazole), analgesics and local anesthetics (aspirin) can cause hemolysis in patients with reduced glucose-6-phosphate dehydrogenase activity [6]. Thus, it is highly desirable to use alternative intervention methods to treat hepatic fibrosis.

In traditional Chinese medical science, hepatic fibrosis can be attributed to the deficiency of the liver-yin and kidney-yin, which perform blood stasis. Traditional Chinese Medicines (TCMs) with novel characteristics (*i.e.*, multi-ingredient and multi-target) [7] have been used for treating liver disease, including hepatic fibrosis, for thousands of years. Jiaqi Ganxian Granule (*JGG*) was developed by Hunan Jiuzhitang Co., Ltd. (Hunan, China) and Hunan University of Chinese Medicine. This prescription was used for the treatment of liver for several tens of years in clinical experience, which consists of about ten components: *Radix Paeoniae Rubra*, *Carapax Trionycis*, *Radix Achyranthis Bidentatae*, *Semen Persicae*, *Rhizoma Curcumae* and *Eupolyphaga sinensis Walker* have the function of activating the blood and eliminate stasis; *Cortex Magnoliae Officinalis* and *Rhizoma Corydali*s are responsible for smoothing liver to recuperate qi; and *Radix Astragali seu Hedysari* and *Poria cocos* act to strengthen spleen to regulate qi. Table 1 shows the main active components of *JGG* [8,9,10,11,12,13,14,15,16,17,18,19,20,21,22,23,24,25,26,27,28,29,30,31,32]. Based on the TCM concept, we predicted that *JGG* would have a therapeutic effect for treating liver fibrosis with almost no side effects. However, due to the complex composition of *JGG*, the therapeutic mechanism is still poorly understood.

Although TCMs have an obvious therapeutic effect, the complicated compositions and unidentified mechanisms may limit the acceptance of TCMs by Western consumers. At present, researchers have found that TCMs can reduce liver fibrosis symptoms and protect liver function through an anti-inflammatory response, anti-proliferation and activation of hepatic stellate cells to reduce collagen synthesis and promoting ECM degradation [33,34,35,36,37]. Some progress had been made, but the molecular mechanisms underlying the therapy of TCMs at the systemic level still urgently need to be understood. Such an understanding will facilitate the rational use of TCMs in the subsequent clinical therapy.

The metabolomics strategy can globally analyze small compounds contained in biological samples and help to understand the pathophysiology [38]. Meanwhile, Ultra-Performance Liquid Chromatography-Time-Of-Flight Mass Spectrometry (UPLC-TOF-MS) has become more popular for the direct identification of multiple components for the quality control of TCMs [39]. Recently, the therapeutic mechanisms of some TCMs have been studied using the UPLC-TOF-MS-based metabolomics strategy extensively; for example, the potential biomarkers in liver injury and the hepatoprotective effects of yinchenhao [40]; the therapy effect of scoparone against alcohol-induced hepatotoxicity was also evaluated [41]. These results indicate that the metabolomics strategy can provide a strong platform for unraveling the molecular mechanisms of TCMs’ treatment. Hepatic metabolic changes induced by oral medicine including *JGG* could be directly reflected in the blood that flows through the liver and detected by UPLC-TOF-MS for the evaluation of the TCMs’ protection against liver fibrosis.

Animal models of human diseases are useful tools in the study of pathogenic processes [42]. A reliable animal model of disease is clinically relevant to humans, could be used for the elucidation of the mechanism of disease pathogenesis [43] and diagnostic biomarker discovery. Small animals, including rats, are very useful for easy management and present minimal logistical, financial or ethical problems [44]. To clarify the therapeutic mechanism, the hepatic fibrosis model has been successfully induced by carbon tetrachloride (CCl_4_) in rats [45]. The rat hepatic fibrosis model can mimic the liver damage occurring in the human body, thereby obviating clinical sampling.

In this work, the CCl_4_-induced hepatic fibrosis rat model was constructed and treated with *JGG*. The UPLC-TOF-MS-based metabolomics strategy was performed to evaluate whether this technique could provide us with some insights into the therapeutic mechanism of *JGG* treatment of hepatic fibrosis.

## 2. Results and Discussion

### 2.1. Serum Markers Tests

**Figure 1:**
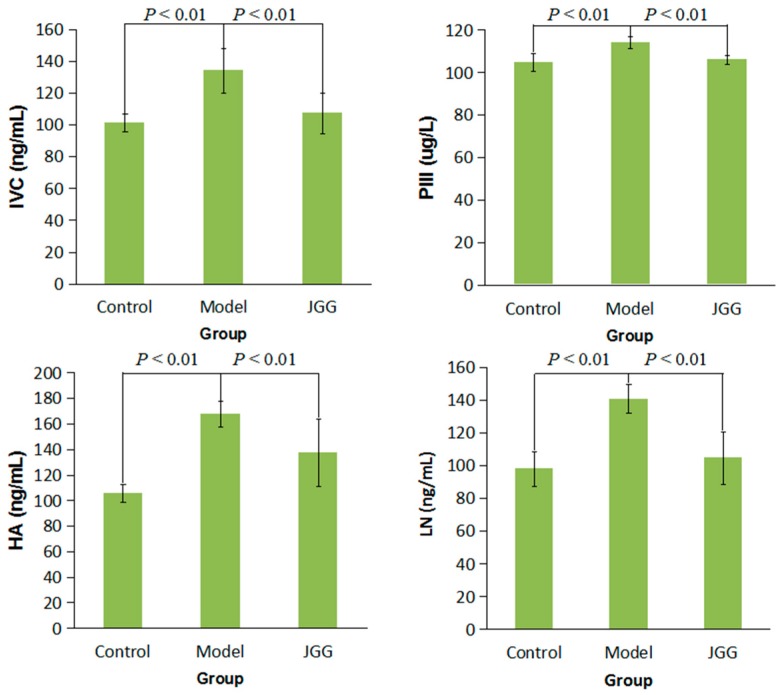
Effect of *JGG* on serum levels of CIV, PCIII, HA and LN in CCl_4_-induced liver fibrosis rats. Value are expressed as $\bar{x}\;\pm \;S$.

As shown in Figure 1, the levels of Collagen Type IV (CIV), Procollagen III (PCIII), Hyaluronic Acid (HA) and Laminin (LN) in serum were significantly increased in the model group compared to the control group (*p* < 0.01), while the contents of CIV, PCIII, HA and LN were all significantly decreased in the *JGG* group compared to the model group (*p* < 0.01). It has been reported that astragaloside IV of *Radix Astragali seu Hedysari* in *JGG* can suppress collagen production of activated hepatic stellate cells via the oxidative stress-mediated p38 MAPK pathway [36], and the bioactive peptide of *Carapax Trionycis*, which is an active compound of *JGG*, has the function of eliminating ECM [30].

### 2.2. Histological Changes Induced by CCl_4_

**Figure 2:**
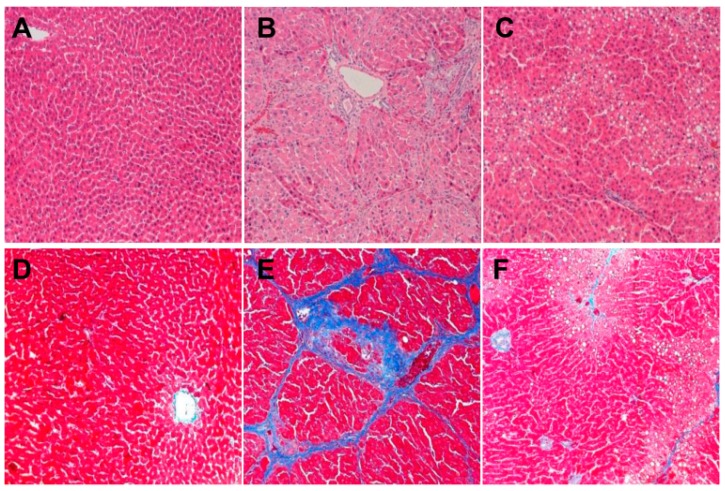
Liver histological changes induced by CCl_4_ overload and the effects of *JGG* treatment in rats. Representative microscopic photographs (original magnification: ×100) were obtained from: (**A**) HE staining of the control group; (**B**) HE staining of the CCl_4_ model group; (**C**) HE staining of the *JGG* intervention group; (**D**) Mallory staining of the control group; (**E**) Mallory staining of the CCl_4_ model group; (**F**) Mallory staining of the *JGG* intervention group.

Hematoxylin and Eosin (HE) staining of liver was observed for histological changes. Rat liver cells in the control group arranged by rules, and the cell structure was clear and had a normal lobular architecture with central veins and radiating hepatic cords (Figure 2A). The collagen fibers increased and were connected to form pseudolobules, which demonstrated the successful establishment of the hepatic fibrosis model in rats (Figure 2B). The degree of liver fibrosis observed in the *JGG* group was apparently low, and no obvious pseudolobules were detected (Figure 2C).

### 2.3. Collagen Change Induced by CCl_4_

Mallory staining was employed to visualize the localization of collagen fibers (bright blue) within the context of the liver architecture (dark purple and pink). The collagen of tissues in the control group was less, only found around the central vein (Figure 2D); the liver tissue of rats in the model group showed a large number of cross-linked collagen fibers (Figure 2E); the collagen of the *JGG* (Figure 2F) intervention group was markedly reduced; the distribution of a small amount of collagen was observed only in the portal area, central vein and interlobular area.

### 2.4. Metabolic Changes in the Model and Drug Intervention Groups

**Figure 3:**
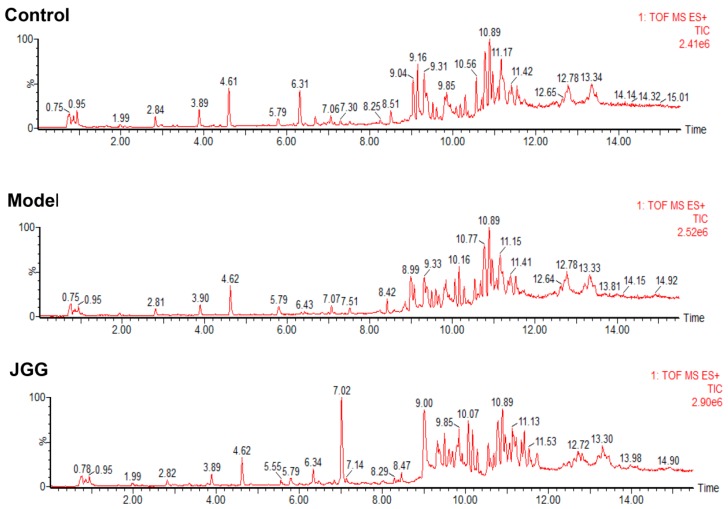
Representative positive TIC chromatograms of serum obtained from the control, model and *JGG* intervention groups. (**A**) Control group; (**B**) CCl_4_ model group; (**C**) *JGG* intervention group.

A greater number of serum metabolites of the UPLC-TOF-MS was found by the analysis in ESI+ mode than in ESI-mode. Representative positive Total Ion Current (TIC) chromatograms of rat serum obtained from the control, model and *JGG* intervention groups are shown in Figure 3. The chromatograms can reveal obvious metabolic differences among the three groups. The reproducibility of the data by the UPLC-TOF-MS method was demonstrated, and the relative standard deviations (RSD %) of the retention time, *m*/*z* and peak area are listed in Table S1 in the Supplementary Materials. The RSDs of the retention time, *m*/*z* and peak area variations for the representative compounds were lower than 0.12%, 0.002% and 5.0%, respectively, demonstrating that the method was reproducible for the metabolic study.

**Figure 4:**
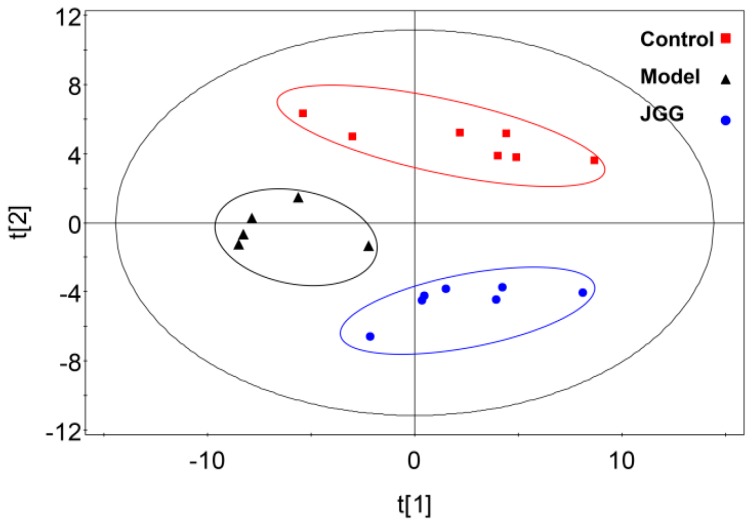
Score plots from the PCA model derived from the UPLC-MS profile of serum obtained from rats. 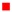: control; 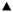: model; 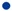: *JGG*. The ellipses are only for easier data visualization.

More subtle changes were determined using a pattern recognition approach, such as Principal Component Analysis (PCA) and Partial Least Square Discriminant Analysis (PLS-DA). The result showed significant separation in the score plot of PCA among the three groups (Figure 4). Distinct clustering between the control group and the model group suggested that CCl_4_ can lead to apparent changes of rat endogenous metabolites. Moreover, the *JGG* intervention group lay between the control and model group on the scores plot. The results of PCA illustrated the profile changes in the serum of rats in each group; the changes and metabolic pathway involved specific metabolites in the CCl_4_ model process, and *JGG* adjusted the differences in the metabolic pathway for the intervention effects on liver fibrosis.

**Figure 5:**
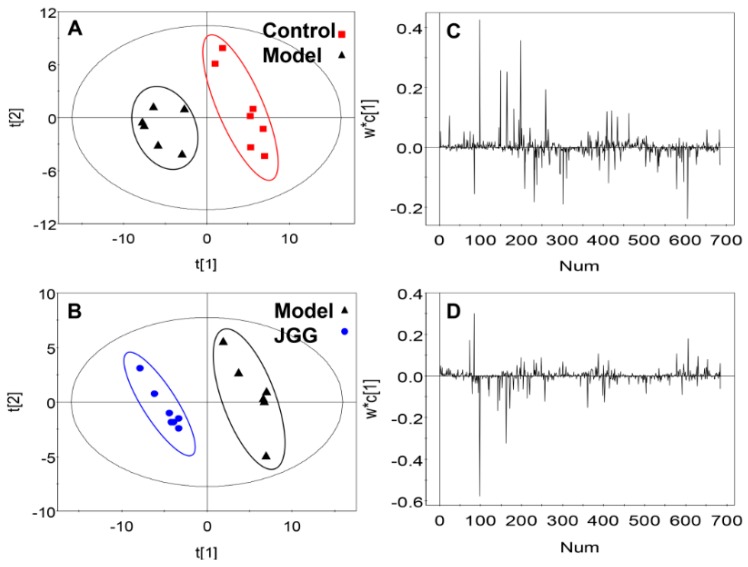
Score plot of the pairwise comparison between (**A**) the control *vs.* the CCl_4_ model group. R2X (cum) = 0.494, R2Y (cum) = 0.928, Q2 (cum) = 0.725; (**B**) *JGG vs.* the CCl_4_ model group. R2X(cum) = 0.463, R2Y (cum) = 0.974, Q2 (cum) = 0.827. Loading plot of the pairwise comparison between (**C**) the control *vs.* CCl_4_ model group and (**D**) *JGG vs.* the CCl_4_ model group. NUM is the abbreviation of Number. The ellipses are only for easier data visualization.

In order to obtain a higher level of separation between groups, PLS-DA was applied, which contributed to further determining the relevant differences in the metabolites (Figure 5). The control group was separated from the model group along the t [1] axis (Figure 5A). Meanwhile, the *JGG* group can be separated from the model group along the t [1] axis (Figure 5B).

**Figure 6:**
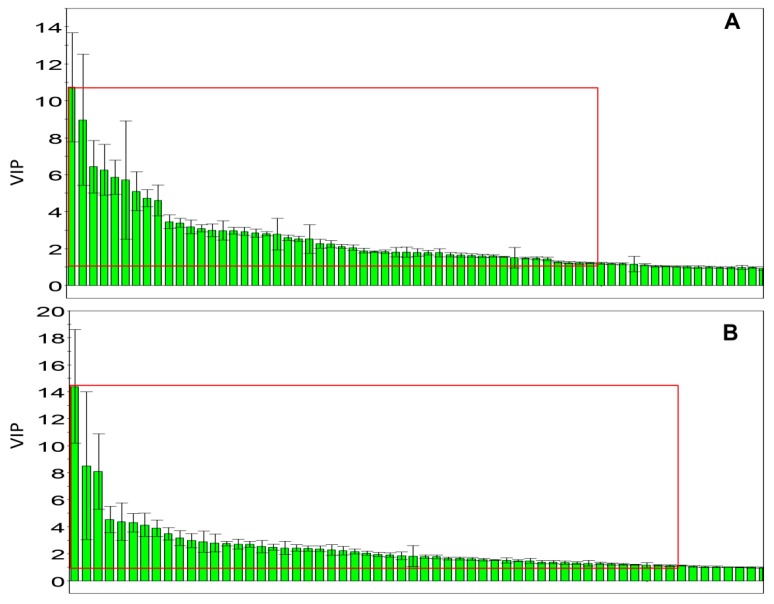
Variable importance projection plots for the serum metabolites along the component. (**A**) Control *vs.* the CCl_4_ model group using PLS-DA; (**B**) *JGG vs.* the CCl_4_ model group using PLS-DA.

The loading plots and Variable Importance Projection (VIP) were constructed from PLS-DA analysis. The absolute value of W*C of the peaks was more than 0.05 (Figure 5C,D), and the value of VIP exceeded 1.00 (Figure 6); these were selected as candidate markers and thought to reflect the metabolic characteristics of the study. The data obtained from three experimental groups were imported into Statistical Product and Service Solutions (SPSS) 17.0 to examine the statistical significance of the differences of the markers by Welch *t*-tests, in order to figure out the potential markers; the data with *p* < 0.05 were deemed to be significant.

In order to study the different compounds and the changes of the biochemical pathways in the metabolic network, the exact high-resolution molecular mass data from redundant *m*/*z* peaks, which correspond to the formation of different parent and product ions, were used to identify the molecular mass of the metabolites. The molecular weight tolerance was set at 0.01 Da when searching in the Human Metabolome Database (HMDB) database (http://www.hmdb.ca/), Metlin (https://metlin.scripps.edu/index.php), Chemspider and using the Kyoto Encyclopedia of Genes and Genomes (KEGG) (http://www.genome.jp/kegg/) to figure out the metabolic pathway.

According to the screening criteria of the above analysis, 17 differences were searched and tentatively identified in metabolites to distinguish the serum metabolic profile in the control group and the model group, which can be a systematic characterization of rat liver fibrosis physiological and pathological changes.

These variables can be regarded as potential marker metabolites to provide the basis for clinical diagnosis and further the drug treatment mechanism of hepatic fibrosis (Table 2), which are mostly related to the creatine pathway, sphingolipid metabolism, glycerophospholipid metabolism, fat digestion and absorption, *N*-acylethanolamine biosynthesis and arachidonic acid metabolism.

**Figure 7:**
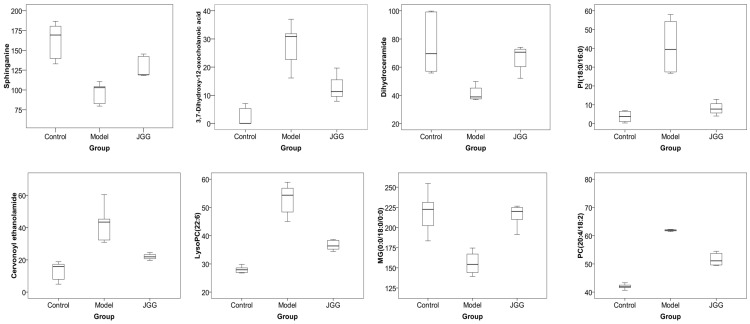
The box plots of eight drug target markers.

**Figure 8:**
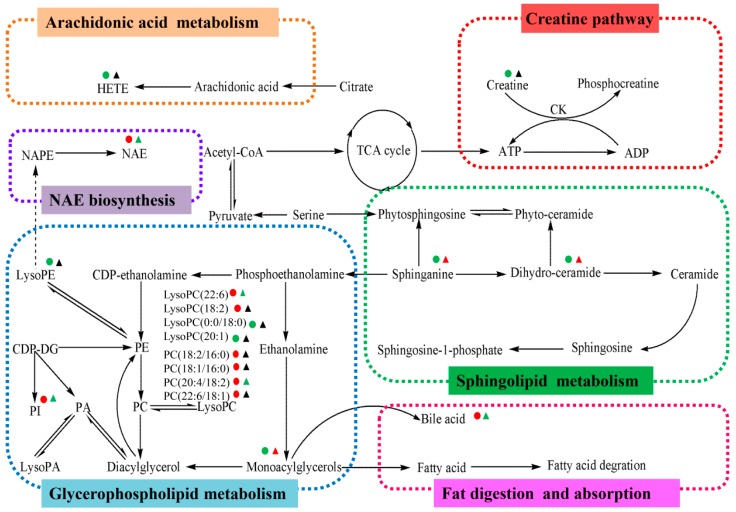
Metabolic pathway of CCl_4_-induced liver fibrosis and the anti-fibrotic effects of *JGG*. Content change tendencies are expressed according to the color: red, significant increases (*p* < 0.05); green, significant decreases: (*p* < 0.05); black, no significant changes (*p* > 0.05). The model group compared to the control group; the *JGG* group compared to the model group.

Furthermore, *JGG* can significantly alter the concentration of eight different metabolites to a normal trend (Table 3), and the content trend of each potential marker is shown by a box plot (Figure 7). This suggested that *JGG* prevented hepatic fibrosis by regulating glycerophospholipid metabolism, sphingolipid metabolism, fat digestion and absorption and N-acylethanolamine biosynthesis; the metabolic pathways of different metabolites are summarized in Figure 8.

#### 2.4.1. Creatine Pathway

Creatine (Cr) can be synthesized by the liver endogenously. Creatine kinase, catalyzing the reversible transfer of the *N*-phosphoryl group from phosphocreatine to adenosine diphosphate (ADP) for regenerating adenosine triphosphate (ATP), is a major enzyme of higher eukaryotes that deals with high and fluctuating energy demands to maintain energy homeostasis and stability in cells [46]. In this study, CCl_4_ injection decreased the creatine content in comparison to the control group; we infer that CCl_4_ injured the liver cell and might lower the expression or the activity of creatine kinase, so that it suppressed the generation of creatine in the body and reduced the ATP supply for energy metabolism. While *JGG* did not affect creatine as expected, even so, it can be a diagnostic target of hepatic fibrosis in the future.

#### 2.4.2. Arachidonic Acid Metabolism

Furthermore, arachidonic acid metabolism was disturbed by CCl_4_ in the model group. Arachidonic acid can be oxygenated and converted to hydroxyeicosatetraenoic acids (HETEs) by different enzymes, including lipoxygenases, cyclooxygenases and cytochrome P450s [47]. HETEs can activate peroxisome proliferator-activated receptors alpha (PPARα), which plays an important role in regulating lipid metabolism [48]. In this study, reduced 8-HETE in the model group might contribute to the turbulence of lipid metabolism induced by CCl_4_. Despite that *JGG* did not act on HETE, it can be regarded as a different metabolite for further diagnosis.

#### 2.4.3. Glycerophospholipid Metabolism

Rats treated by CCl_4_ can be led to the disorder of the metabolism of lipids to induce hepatic fibrosis. Glycerophospholipids (GPs) are amphipathic molecules and can function as integral membrane proteins, transporters, receptors and ion channels and as a storage deposit for lipid mediators [49]. In this study, the significant content change of phosphatidylcholines (PCs) in the CCl_4_ treatment group suggested that the glycerophospholipid metabolism process of liver fibrosis might be disturbed. In malignant tissues, enhanced synthesis of PCs might be attributed to the increase of lysophosphatidylcholine acyltransferase (LPCAT) activity, leading to fatty acid remodeling via the deacylation-reacylation cycle [50]. It has been reported that PCs synthesis can be enhanced by alcohol stimulation and plays a protective role in resisting alcohol liver injury [51]. Similarly, in this study, the content of PC (18:2/16:0), PC (18:1/16:0), PC (20:4/18:2) and PC (22:6/18:1) increased in the model group induced by CCl_4_ to protect the liver from injury. While the content of PC (20:4/18:2) decreased to normal levels in the *JGG* group, it might illustrate the recovery effect of *JGG*.

Lysophospholipids are metabolites of GPs metabolism and can be transiently generated during the remodeling of GPs [52]. Compared to the control group, serum lysophosphatidylcholine (LysoPC) in the model group varied largely. The contents of serum saturated or monounsaturated LysoPCs (LysoPC (18:0) and LysoPC (20:1)) decreased, whereas polyunsaturated LysoPCs (LysoPC (22:6) and LysoPC (18:2)) content increased. Such results were consistent with what were reported previously [53]. The significant changes of LysoPCs may result from different mechanisms. First of all, LysoPCs are generated in liver tissues from hydrolysis of the sn-2 fatty acyl bond of phospholipids by phospholipase A_2_ (PLA_2_). CCl_4_ treatment can promote PLA_2_’s activity in liver cancer, which has been reported before [54], as a result leading to the augment of LysoPCs. On the other hand, LysoPCs can be generated by granulocytes by the release of reactive oxygen species (ROS); this may induce extensive oxidative damage by CCl_4_, particularly on lipids in areas where inflammation occurs, so it is reflected by the changes of LysoPCs, which are the breakdown products of PC [55]. Elevated hepatic concentrations of various LysoPCs have been also reported for humans who have steatotic *vs.* non-steatotic livers [56]. While in this study, the content of LysoPC (22:6) decreased to a normal level in the *JGG* intervention group, this showed that the *JGG* used in this experiment can lower the activity of PLA_2_ and reduce inflammation and oxidative damage to produce the therapeutic effect for liver fibrosis. Similarly, it has been reported that biphenyl-type neolignans, one of the active compounds of *JGG*, are a natural component of *Magnolia officinalis* and have the effect of reducing the release of ROS and, as a result, inhibiting the pathogenesis of inflammation [24], and paconiflorin of *Radix Paeoniae Rubra* in *JGG* has the function of reducing inflammatory reactions in liver [33].

However, interestingly, enhanced conversion of LysoPCs to lysophosphatidic acids (LysoPAs) by lysophospholipase D may reduce serum saturated or monounsaturated LysoPCs [57]. This may explain the decrease of LysoPC (18:0) and LysoPC (20:1). Moreover, lysophosphatidyl ethanolamine (LysoPE) (22:0/0:0) content was downregulated in the CCl_4_ treatment group to suppress the degradation of phosphatidylethanolamine (PE). Although LysoPE (22:0/0:0), LysoPC (18:0) and LysoPC (20:1) were not adjusted by *JGG*, they can be regarded as different metabolites for further diagnose.

Even though only a very minor portion of the cellular lipid content was identified, phosphoinositides (PI) and its phosphorylated species are still paramount signaling molecules [58]. While PI (18:0/16:0) content increased in rats with liver fibrosis, in the *JGG* intervention, the PI content decreased to a normal trend.

The results above reveal that CCl_4_ may lead to metabolic disorders of glycerophospholipid metabolism, which can contribute to hepatic fibrosis. *JGG* had the function of balancing the disorders by the antioxidant effect to relieve the inflammatory reaction and repair the damaged liver cell membrane.

#### 2.4.4. Sphingolipid Metabolism

There also was turbulence in the sphingolipid metabolism in rats that had liver fibrosis. Sphingolipids are a diverse class of lipids that are composed of free sphingoid bases and their phosphates, ceramides and sphingomyelins, as well as complex glycosphingolipids [59]. Many of these lipids are well-known signaling molecules that have been implicated in various diseases, including metabolic disorders [60] and cancer [61].

Ceramides (Cers) involve the inflammatory response; they act as secondary messengers in the signal transduction pathway, which is triggered by several agents of stress, including oxidative stress, ionizing radiation and extracellular stimuli, such as pro-inflammatory cytokines and lipopolysaccharide [62]. Structurally, a ceramide consists of a long-chain sphingoid base, such as sphingosine (So), sphinganine (Sa) and phytosphingosine (PhytoSPH), with an amide-linked fatty acid. In this study, sphinganine and dihydroceramide (Dih-Cer) were all decreased in the serum of rats that had liver fibrosis to produce ceramides as a response to inflammation; theoretically, this suggests that CCl_4_ may involve perturbations of sphingolipid metabolism and the responsible system for inflammation in the hepatic fibrosis model group. It has been reported that carboxymethylation of *Poria cocos* in *JGG* has the effect of liver protection and of an antioxidant [63], as well as the furanodiene of *Rhizoma Curcumae* in *JGG* has a hepatoprotective effect and anti-inflammatory function [30]; moreover, tannins as an important active component of *Radix Paeoniae Rubra*, *Cortex Magnoliae Officinalis* and *Rhizoma Curcumae* in *JGG* have the effect of anti-fibrotic activity on the liver [64]. In this study, *JGG* can regulate the metabolism by supplying the concentration of sphinganine and dihydroceramide at a normal level by reducing inflammation from liver injury.

#### 2.4.5. Fat Digestion and Absorption

Bile Acids (BAs) are endogenous molecules that normally regulate cholesterol homeostasis, lipid solubilization and metabolic signaling [65], which are endogenous markers of liver transport and synthesis function. Altered plasma profiles of BAs’ composition are also reported for many human chronic liver diseases, including Non-Alcoholic Fatty Liver Disease (NAFLD) [66]. 3,7-Dihydroxy-12-oxocholanoic acid as a bile acid increased in the model group and could be the consequence of a higher bile acid pool due to a higher rate of bile acid synthesis, the result of increased peroxisomal and microsomal metabolism or be caused by hepatocellular injury. This suggested that CCl_4_ may lead to metabolic disorders of fat digestion and absorption metabolism, which can contribute to hepatic fibrosis, while *JGG* reduced the content of BAs to a normal trend. This revealed that they can regulate the chaos of BAs by inhibiting the synthesis of BAs, adjusting peroxisomal and microsomal metabolism, reducing the injury by CCl_4_ to control the progress of hepatic fibrosis.

Diacylglycerol (DG), which is generated by the dephosphorylation of PA, as a source for PC, PE and PS synthesis, can form in the lysosomes of liver by the action of lysosomal phospholipase C on diacylphosphoglycerides [67], while monoacylglycerols (MGs) are formed biochemically via the release of a fatty acid from DG by DG lipase or a hormone-sensitive lipase. MG is broken down by monoacylglycerol lipase. In this study, MG was down regulated in the model group to supply BAs, while *JGG* can balance metabolism.

#### 2.4.6. *N*-Acylethanolamine Biosynthesis

Cervonoyl ethanolamide is *N*-acylethanolamines (NAEs). NAEs are released from *N*-acylphosphatidyl ethanolamines (NAPEs) by phospholipase d-type hydrolases in response to a variety of stimuli. Transient NAE release and accumulation have been attributed to a variety of biological activities, including membrane protection, energy metabolism and immunomodulation in animals [68,69]. In this study, cervonoyl ethanolamide increased in the model group; this suggested that CCl_4_-induced liver injury, which stimulated the body to release NAE to protect the membrane of hepatocytes. Administration of *JGG* inhibited the generation of NAEs and the accumulation of NAPEs, which have also been reported to have cytoprotective functions by three approaches. First, they inhibit the process of necrosis in the individual injured cell; besides, they stimulate the injured cell or adjacent cells to activate the apoptotic process, which contributes to stopping the spreading of necrosis; moreover, they suppress the release of mediators that promote necrosis and inflammation [70]; finally, they inhibit the process of hepatic fibrosis induced by CCl_4_.

**Figure 9:**
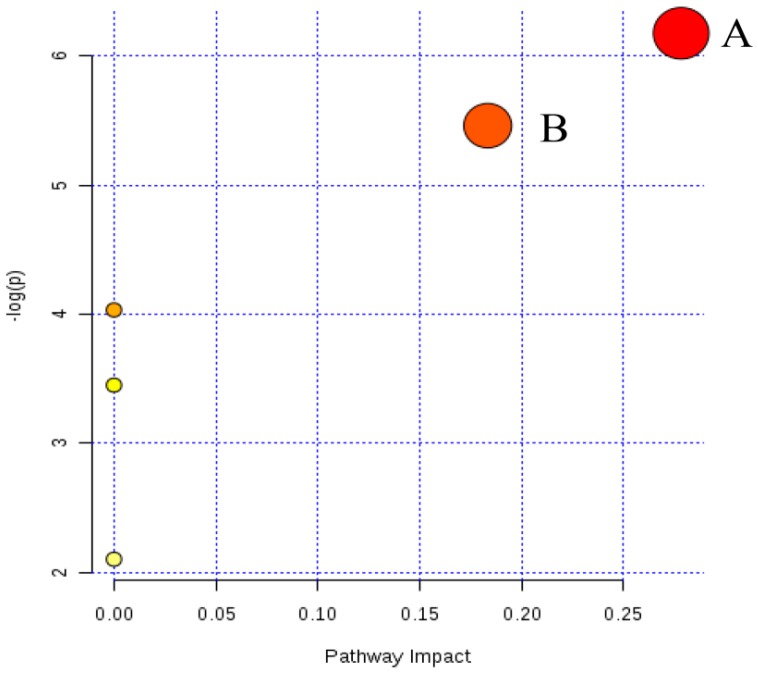
Summary of the pathway analysis with MetPA. (**A**) Glycerophospholipid metabolism; (**B**) sphingolipid metabolism.

Furthermore, the most relevant pathways were analyzed by the metabolic pathway analysis (MetPA) on MetaboAnalyst 3.0. The impact value and −log(*p*) of the results were used out to evaluate the importance of the pathways on the inhibitory effects of *JGG* on hepatic fibrosis (Figure 9). Both glycerophospholipid metabolism and sphingolipid metabolism were identified as relevant pathways from pathway topology analysis (impact > 0.01). While glycerophospholipid metabolism has a better −log(*p*) value (Figure 9A,B). Therefore, glycerophospholipid metabolism was recognized as the most relevant pathway in the inhibitory effects of *JGG* on hepatic fibrosis.

## 3. Materials and Methods

### 3.1. Chemicals and Reagents

CCl_4_ was obtained from China National Pharmaceutical Group Corporation (Beijing, China). The solvents acetonitrile (ACN) and methanol were of HPLC grade from Merck (Darmstadt, Germany). Formic acid with a purity of 96% was purchased from Tedia (Fairfield, OH, USA). Pure water (18 MΩ) was prepared using the Milli-Q system (Millipore, Milford, MA, USA). PCIII, CIV, LN and HA kits were obtained from Beijing Sino-uk institute of Biological Technology (Beijing, China). Other reagents and chemicals were of analytical grade.

### 3.2. JGG Preparation

*JGG* extract was provided by Hunan Jiuzhitang Co., Ltd. (code number approved by China Food and Drug Administration: Z20030056), before producing granules with the initial concentration of 1 g/mL, which was stored in the cold for preservation until use. The extract was diluted to 0.02 g/mL for treatment.

### 3.3. Animals and Grouping Design

Adult male Sprague-Dawley (SD) rats weighing 110–130 g were purchased from Beijing Vital River Laboratory Animal Technology Co., Ltd. (Beijing, China), with the confirmation number SCXK (Jing) 2012-0053. The animals were acclimatized to the facilities in cages (23 °C, 12 h/12 h light/dark, 50% humidity, *ad libitum* access to food and water) for 7 days prior to experimentation. The experiments were carried out in accordance with the Guidelines for Animal Experimentation of Beijing University of Chinese Medicine (Beijing, China). The principles had been approved by the Animal Ethics Committee of this institution.

SD rats were randomly divided into the following three groups: Group A, control group (*n* = 7); Group B, CCl_4_-treated model group (*n* = 7); Group C, *JGG* intervention group (*n* = 7). Hepatic fibrosis was induced with the protocol described below: Groups B and C received CCl_4_ (1 mL/kg 40% CCl_4_, diluted in olive oil) by intraperitoneal injection (i.p.), twice weekly for 6 weeks. The *JGG* treatment was started with the concentration of 0.02 g/mL at the first dose of 40% CCl_4_, and the dosage by oral administration was performed once a day by gavage at 10 mL/kg for 6 weeks.

### 3.4. Samples Collection

At the end of the sixth week, 21 rats were anesthetized with 10% chloral hydrate, and blood was obtained from the abdominal aorta for serum metabolic analysis. Blood samples were centrifuged at 3000 rpm for 20 min to obtain serum and stored at −80 °C until analysis. Livers were isolated and stored at −80 °C until use for pathological observation.

### 3.5. Analysis of Serum Markers

PCIII, CIV, LN and HA were determined to measure the degree of ECM degradation by radioimmunoassay kits according to the manufacturer’s instructions.

### 3.6. Histopathological Analysis

A portion of each liver tissue was fixed in 10% paraformaldehyde and embedded in paraffin, then cut into 4 μm-thick sections for histomorphological analysis. After drying, liver tissue was stained with HE staining for histological observations and Mallory staining to visualize collagen deposition.

### 3.7. Metabolomics Analysis

#### 3.7.1. Serum Sample Preparation

Two hundred-microliter serum samples were diluted at a ratio of 1:4 with methanol-acetonitrile (*v*:*v* = 4:1), put into micro-centrifuge tubes, vortexed for 1 min, followed by being centrifuged at approximately 12,000 rpm, 4 °C for 15 min. The supernatant was placed in another micro-centrifuge tube, with nitrogen drying, dissolved again by 200 μL pure water and vortexed for 1 min, then centrifuged at 12,000 rpm, 4 °C for 15 min. The obtained supernatant was injected into the UPLC/Q-TOF MS system for metabolite analysis.

#### 3.7.2. UPLC-MS Analysis

UPLC/MS and UPLC/MS/MS experiments were performed on the Waters ACQUITY UPLC^®^ and Xevo G2 Q-Tof MS systems (Waters Corporation, Milford, MA, USA) equipped with an electrospray ion source and a hybrid Quadrupole-Time-Of-Flight (Q-TOF) mass spectrometer in the MS^E^ model. The system was controlled with the MarkerLynx applications manager Version 4.1 (Waters Corp., Manchester, UK). The Acquity UPLC HSS T3 column (2.1 mm × 100 mm, 1.8 μm, Waters, Milford, MA, USA) was used for the analyses. The mobile phase consisted of 0.1% formic acid water (Solvent A) and methanol (Solvent B).

The serum samples were analyzed using a gradient elution at a flow rate kept at 0.3 mL/min, and the injecting volume was set at 1.50 μL. The gradient program is shown as follows: 0–1 min: 100% A + 0% B; 1–7 min: 40% A + 60% B; 7–10 min: 0% A + 100% B; 10–15 min: 0% A + 100% B; 15–18 min: 100% A + 0% B.

High purity nitrogen (N_2_) was used as the nebulizer and auxiliary gas; argon (Ar) was utilized as the collision gas. TOF/MS analysis worked using full scan mode, and the mass range was set at 50–1200 *m*/*z*. The Q-TOF mass spectrometer was operated with electrospray ionization (ESI) in positive and negative ion mode with a capillary voltage of 3 kV, a sampling cone voltage of 35 kV, a cone gas flow of 50 L/h, a desolvation gas flow of 750 L/h, a desolvation temperature of 350 °C, a source temperature of 110 °C and a collision energy of 6.0 eV.

Mass accuracy was maintained by using a LockSpray™. Leucine enkephalin (0.2 ng/mL infused at 5 μL/min) was used as a reference lock mass, generating a reference ion at 556.2771 Da ([M + H]^+^) and 554.2187 Da ([M + H]^−^). The LockSpray scan time was set at 0.5 s with an interval of 15 s, and data were averaged over three scans. MS and MS/MS data were acquired using two interleaved scan functions in the MS^E^ mode. The first scan function was set at 6 eV in order to collect information on the intact precursor ions in the sample, and the second scan function was ramped from 15 eV–35 eV to obtain the fragment ion data from the ions in the preceding scan.

#### 3.7.3. Analytical Method Validation

To ensure the reproducibility of the UPLC-TOF-MS method, the repeatability was validated based on the analysis of quality control (QC) samples. QC was prepared by pooling the same volume from each serum together, which was then pretreated in the same way as the samples. The repeatability of the method was evaluated using 6 replicates of the QC sample, which were inserted into the analytical batch randomly.

#### 3.7.4. Data Analysis

The raw UPLC/MS data processing was carried out with the Masslynx V 4.1, in order to identify potential discriminant variables. For the peak finding, peak alignment and peak filtering of the raw data, the parameters used were as follows: initial retention time 0.50 min, final retention time 20.00 min, mass range 50–1200 *m*/*z*, mass tolerance 0.05 Da; the noise elimination level was set at 3.00. A list of the ion intensities of all components with their corresponding retention time and *m*/*z* as the identifier was generated. Preprocessed data were then exported to SIMCA-P 11.0 (Umetrics, Umea, Sweden) and used to construct multivariate statistical models via PCA and PLS-DA.

After the analyses, the ion spectra of potential biomarkers were matched with the structural data of metabolites acquired from the HMDB, Metlin and Chemspider for biomarker identification. Furthermore, the metabolic pathway was figured out using the KEGG.

## 4. Conclusions

In this study, the CCl_4_-induced hepatic fibrosis model was constructed in SD rats to investigate the anti-fibrosis effects of *JGG*. The UPLC-TOF-MS-based metabolomics strategy with multivariate analysis was performed to unravel the molecular mechanisms underlying *JGG* treatment. The results indicated that *JGG* has an efficient effect of inhibiting the progression of liver fibrosis in rats. The mechanism of action of *JGG* involves modulation, including glycerophospholipid metabolism, sphingolipid metabolism, fat digestion and absorption, as well as *N*-acylethanolamine biosynthesis. The inference confirmed that the effect of *JGG* was generated by a multicomponent synergy function, while glycerophospholipid metabolism played vital roles in the inhibitory effects of *JGG* on hepatic fibrosis according to MetPA analysis. The therapeutic effects of *JGG* may be attributed to the anti-inflammatory function and inhibition of oxidative stress in hepatic fibrosis.

## Figures and Tables

**Table 1:** The information of the main active components of *JGG*.

Herb	Active Component	Ref.
*Radix Paeoniae Rubra*	Paeoniflorin, Albiflorin, Benzoic Acid, Tannins, Triterpenoids, Flavonoids, Phenolic Acids	[8,9,10,11]
*Radix Achyranthis Bidentatae*	Polysaccharides, Ketosteroids, Triterpenoids	[12,13,14]
*Poria cocos*	Pachymic acid, Eburicoic acid, Dehydrotumulosic Acid, Polysaccharide, Heteropolysaccharides	[15,16,17]
*Semen Persicae*	Amygdalin, Botanical, Glycoside, Amygdalin, Lipids	[18,19]
*Rhizoma Corydalis*	Corydaline, Glaucine, Canadine, Protopine, Tetrahydropalmatine	[20,21]
*Cortex Magnoliae Officinalis*	Biphenols, Polyphenols, Tannins, Magnolol, Honokiol, Neolignans	[22,23,24]
*Radix Astragali seu Hedysari*	Isoflavonoids, Astragalosides, Saponins, Polysaccharides	[25]
*Rhizoma Curcumae*	Tannins, Saponins, Flavonoids, Steroids, Alkaloids, Terpenoids, Curcuminoid, Sesquiterpenoids, Furanodiene	[26,27,28]
*Carapax Trionycis*	Peptide	[29,30]
*Eupolyphaga sinensis Walker*	Vitamins, Essential Amino Acids, Essential Fatty Acids, Isocoumarins, Alkaloid	[31,32]

**Table 2:** The potential markers of the therapeutic approaches for hepatic fibrosis.

No.	RT (min)	Measured MS (*m*/*z*)		Adduct	Error (mDa)	Metabolites	HMDB Formula	Major Metabolic Pathway	Model
		Positive	Negative						
1	0.95	132.0777	130.0623	[M + H]^+^	0.9	Creatine	C_4_H_9_N_3_O_2_	Creatine pathway	↓ ^b^
2	9.51	302.3050	--	[M + H]^+^	0.4	Sphinganine	C_18_H_39_NO_2_	Sphingolipid metabolism	↓ ^c^
3	9.66	429.2609	405.2629	[M + Na]^+^	0.2	3,7-Dihydroxy-12-oxocholanoic acid	C_24_H_38_O_5_	Fat digestion and absorption	↑ ^b^
4	9.85	330.2968	328.2448	[M + H]^+^	3.4	Dihydroceramide	C_19_H_39_NO_3_	Sphingolipid metabolism	↓ ^b^
5	10.17	839.5669	837.5508	[M + H]^+^	2.5	PI (18:0/16:0)	C_43_H_83_O_13_P	Glycerophospholipid metabolism	↑ ^a^
6	10.18	373.2743	--	[M + H]^+^	0.6	Cervonoyl ethanolamide	C_24_H_36_O_3_	*N*-acylethanolamine biosynthesis	↑ ^c^
7	10.56	343.2252	319.2274	[M + Na]^+^	0.8	8-HETE	C_20_H_32_O_3_	Arachidonic acid metabolism	↓ ^a^
8	10.75	568.3404	612.3293	[M + H]^+^	0.6	LysoPC (22:6)	C_30_H_50_NO_7_P	Glycerophospholipid metabolism	↑ ^c^
9	10.77	520.3398	518.2407	[M + H]^+^	0.0	LysoPC (18:2)	C_26_H_50_NO_7_P	Glycerophospholipid metabolism	↑ ^c^
10	11.16	1047.7383	568.3617	[2M + H]^+^	3.4	LysoPC (0:0/18:0)	C_26_H_54_NO_7_P	Glycerophospholipid metabolism	↓ ^a^
11	11.21	572.3705	594.3704	[M + Na]^+^	1.8	LysoPC (20:1)	C_28_H_56_NO_7_P	Glycerophospholipid metabolism	↓ ^b^
12	11.25	560.3708	582.3624	[M + Na]^+^	2.1	LysoPC (22:0/0:0)	C_27_H_56_NO_7_P	Glycerophospholipid metabolism	↓ ^b^
13	11.41	381.2979	--	[M + Na]^+^	0.4	MG (0:0/18:0/0:0)	C_21_H_42_O_4_	Glycerophospholipid metabolism	↓ ^b^
14	12.79	758.5696	802.5616	[M + H]^+^	0.2	PC (18:2/16:0)	C_42_H_80_NO_8_P	Glycerophospholipid metabolism	↑ ^b^
		780.5525		[M + Na]^+^	1.1	PC (18:2/16:0)	C_42_H_80_NO_8_P	Glycerophospholipid metabolism	↑ ^b^
15	13.20	760.5880	804.5800	[M + H]^+^	2.9	PC (18:1/16:0)	C_42_H_82_NO_8_P	Glycerophospholipid metabolism	↑ ^c^
		782.5699		[M + Na]^+^	2.9	PC (18:1/16:0)	C_42_H_82_NO_8_P	Glycerophospholipid metabolism	↑ ^c^
16	12.63	806.5737	850.5636	[M + H]^+^	4.3	PC (20:4/18:2)	C_46_H_80_NO_8_P	Glycerophospholipid metabolism	↑ ^c^
		828.5555		[M + Na]^+^	4.1	PC (20:4/18:2)	C_46_H_80_NO_9_P	Glycerophospholipid metabolism	↑ ^c^
17	13.35	832.5839	830.5999	[M + H]^+^	1.2	PC (22:6/18:1)	C_46_H_84_NO_8_P	Glycerophospholipid metabolism	↑ ^a^

(Compared to the control group, ^a^ *p* < 0.05; ^b^ *p* < 0.01; ^c^ *p* < 0.001). --: this metabolite can not be found in the MS and MS/MS with negative mode.

**Table 3:** Target biomarkers of *JGG* intervention.

No.	Metabolites	Metabolic Pathway	Trend	
			B/A	C/B
1	Sphinganine	Sphingolipid metabolism	↓ ^c^	↑ ^b^
2	3,7-Dihydroxy-12-oxocholan-oic acid	Fat digestion and absorption	↑ ^b^	↓ ^a^
3	Dihydroceramide	Sphingolipid metabolism	↓ ^b^	↑ ^c^
4	PI (18:0/16:0)	Glycerophospholipid metabolism	↑ ^a^	↓ ^a^
5	Cervonoyl ethanolamide	*N*-acylethanolamine biosynthesis	↑ ^c^	↓ ^a^
6	LysoPC (22:6)	Glycerophospholipid metabolism	↑ ^c^	↓ ^c^
7	MG (0:0/18:0/0:0)	Fat digestion and absorption	↓ ^b^	↑ ^c^
8	PC (20:4/18:2)	Glycerophospholipid metabolism	↑ ^c^	↓ ^c^

A: control group; B: model group; C: *JGG* (^a^ *p* < 0.05; ^b^ *p* < 0.01; ^c^ *p* < 0.001).

## Supplementary Materials

Supplementary materials can be accessed at: http://www.mdpi.com/1420-3049/21/6/698/s1.

## Abbreviations

The following abbreviations are used in this manuscript:

Bas	Bile Acids
CDP-Etn	CDP-Ethanolamine
CDP-DG	CDP-Diacylglycerol
Cer	Ceramide
CIV	Collagen Type IV
Cr	Creatine
CK	Creatine Kinase
DG	Diacylglycerol
Dih-Cer	Dihydro-Ceramide
ECM	Extracellular Matrix
Etn	Ethanolamine
FA	Fatty Acid
GPs	Glycerophospholipids
HA	Hyaluronic Acid
HE	Hematoxylin and Eosin
HETEs	Hydroxyeicosatetraenoic Acids
LPCAT	Lysophosphatidylcholine Acyltransferase
LN	Laminin
LysoPA	Lysophosphatidic Acids
LysoPC	Lysophosphatidylcholine
LysoPE	Lysophosphatidyl Ethanolamine
NAFLD	Non-Alcoholic Fatty Liver Disease
NAEs	*N*-Acylethanolamines
NAPEs	*N*-Acylphosphatidyl Ethanolamines
MGs	Monoacylglycerols
PA	Phosphatidic Acid
PC	Phosphatidylcholine
PCIII	Procollagen Type III
PCr	Phosphocreatine
PE	Phosphatidylethanolamine
p-Etn	Phosphoethanolamine
Phyto-Cer	Phyto-Ceramide
PhytoSPH	Phytosphingosine
PI	Phosphoinositides
PLA2	Phospholipase A2
PS	Phosphatidylserine
Q-TOF	Quadrupole-Time-Of-Flight
QC	Quality Control
Sa	Sphinganine
Ser	Serine
So	Sphingosine
S1P	Sphingosine-1-Phosphate
TCMs	Traditional Chinese Medicines
VIP	Variable Importance Projection
