# Supplementary material for: Metabolic Profile Changes of CCl4-Liver Fibrosis and Inhibitory Effects of Jiaqi Ganxian Granule
Source: Molecules. 2016 May 30;21(6):698. doi: 10.3390/molecules21060698 (PMC6273034; doi:10.3390/molecules21060698)
Supplement: Supplementary file 1 [file molecules-21-00698-s001.pdf]

Supplementary Materials: Metabolic Profile Changes of CCl_4-Liver Fibrosis and Inhibitory Effects of Jiaqi Ganxian Granule

Ge Wang, Zehao Li, Hao Li, Lidan Li, Jian Li and Changyuan Yu

Table S1. Reproducibility testing results of the UPLC-TOF-MS profiling method.

NO.	RT (min)	*m/z*	Repeatability		
			RSD (%)$_{RT}$	RSD (%)$_{m/z}$	RSD (%)$_{Peak\ area}$
1	0.95	132.0777	0.0000	0.0001	3.57
2	9.51	302.3050	0.1192	0.0007	2.70
3	9.66	429.2609	0.0747	0.0002	3.09
4	9.85	330.3369	0.0969	0.0002	4.85
5	10.17	839.5669	0.0722	0.0002	5.00
6	10.18	373.2743	0.0722	0.0001	1.41
7	10.56	343.2252	0.0499	0.0001	1.80
NO.	RT (min)	*m/z*	Repeatability		
			RSD (%)$_{RT}$	RSD (%)$_{m/z}$	RSD (%)$_{Peak\ area}$
8	10.75	568.3404	0.0489	0.0001	3.35
9	10.77	520.3398	0.0489	0.0001	2.71
10	11.16	1047.7383	0.0256	0.0002	1.79
11	11.21	572.3705	0.0322	0.0002	3.37
12	11.25	560.3708	0.0937	0.0003	3.96
13	11.41	381.2979	0.0388	0.0001	1.73
14	12.79	758.5696	0.0300	0.0001	1.06
15	12.79	780.5803	0.0300	0.0019	0.99
16	13.20	760.5880	0.0290	0.0003	2.20
17	13.20	782.5699	0.0290	0.0012	2.36
18	12.63	806.5737	0.0286	0.0001	2.02
19	12.63	828.5555	0.0286	0.0009	1.54
20	13.35	832.6090	0.0271	0.0001	1.26
